# Impact of a Measles and Rubella Vaccination Campaign on Seroprevalence in Southern Province, Zambia

**DOI:** 10.4269/ajtmh.20-1669

**Published:** 2021-05-03

**Authors:** Andrea C. Carcelen, Simon Mutembo, Kalumbu H. Matakala, Innocent Chilumba, Gina Mulundu, Mwaka Monze, Francis D. Mwansa, William J. Moss, Kyla Hayford

**Affiliations:** 1 Department of International Health, Johns Hopkins Bloomberg School of Public Health, Baltimore, Maryland;; 2 Ministry of Health, Government of the Republic of Zambia, Lusaka, Zambia;; 3 Macha Research Trust, Choma, Zambia;; 4 Tropical Disease Research Center, Ndola, Zambia;; 5 University of Zambia School of Medicine, Lusaka, Zambia;; 6 University Teaching Hospital, Lusaka, Zambia;; 7 Department of Epidemiology, Johns Hopkins Bloomberg School of Public Health, Baltimore, Maryland

## Abstract

Zambia conducted a measles and rubella (MR) vaccination campaign targeting children 9 months to younger than 15 years of age in 2016. This campaign was the first introduction of a rubella-containing vaccine in Zambia. To evaluate the impact of the campaign, we compared the MR seroprevalence estimates from serosurveys conducted before and after the campaign in Southern Province, Zambia. The measles seroprevalence increased from 77.8% (95% confidence interval [CI], 73.2–81.9) to 96.4% (95% CI, 91.7–98.5) among children younger than 15 years. The rubella seroprevalence increased from 51.3% (95% CI, 45.6–57.0) to 98.3% (95% CI, 95.5–99.4). After the campaign, slightly lower seroprevalence remained for young adults 15 to 19 years old, who were not included in the campaign because of their age. These serosurveys highlighted the significant impact of the vaccination campaign and identified immunity gaps for those beyond the targeted vaccination age. Continued monitoring of population immunity can signal the need for future targeted vaccination strategies.

Zambia conducted a national measles and rubella (MR) vaccination campaign targeting children 9 months to younger than 15 years of age in September 2016. This campaign marked the first use of a rubella-containing vaccine (RCV) in the public sector, and the combined MR vaccine is now included in the routine immunization program.^1^

The impact of a vaccination campaign can be assessed through changes in vaccination coverage or population immunity before and after the campaign. Campaign vaccination coverage was estimated from administrative data from each district or by conducting a community-based vaccination coverage survey. However, vaccination coverage estimates can be difficult to interpret because they do not account for vaccine efficacy and may be based on inaccurate numerators and denominators.^2^ Tracking the numbers of MR cases reported before and after a campaign can also be performed to evaluate campaign effectiveness if surveillance is sufficiently sensitive to identify cases. Zambia’s case-based surveillance system for measles does not meet the World Health Organization’s targets for performance indicators of a sensitive surveillance system, however.^3^

Serological surveillance provides a more direct measure of changes in population immunity before and after a vaccination campaign.^4,5^ Because 92% to 94% population immunity is needed to interrupt measles virus transmission, a serosurvey can determine whether this threshold has been met to achieve measles elimination goals.^6^ Serosurveillance can also identify immunity gaps across wide age ranges before outbreaks occur.^7^

To evaluate the impact of the 2016 MR vaccination campaign, we compared the MR seroprevalence estimates using two serosurveys conducted in Southern Province, Zambia before and after the campaign. The pre-MR campaign serosurvey was conducted using a national biorepository of plasma and dried blood spot specimens for those younger than 2 years generated from the Zambia Population HIV Impact Assessment, a provincially representative, cross-sectional HIV serosurvey.^8^ In 2018, the Zambian National Regulatory Authorization provided authorization to access the biorepository of samples collected between March and August 2016, before the MR vaccination campaign. Ethical approval was also obtained from Johns Hopkins University and Tropical Disease Research Center. We selected a subsample of participants based on their age category (6 months–5 years, 5–9 years, 10–14 years, 15–19 years, and 20–49 years), HIV status, and cluster from the 3566 specimens from Southern Province to test for anti-measles virus and anti-rubella virus IgG antibodies using an indirect enzyme immunoassay (EIA; Euroimmun, Lübeck, Germany) to estimate age-specific seroprevalence at the provincial level.

The post-MR campaign serosurvey was conducted 2 months after the campaign in November 2016, as part of the postcampaign vaccination coverage evaluation survey in Southern Province, Zambia. This cross-sectional community survey followed the sampling strategy of the postcampaign vaccination coverage evaluation survey designed to estimate the proportion of children vaccinated during the campaign.^9^ This nested serosurvey collected dried blood spots obtained using a finger prick from all members of a selected household 9 months of age or older in 14 of the 26 clusters to estimate age-specific seroprevalence in the province for children eligible for the vaccination campaign and adults beyond the age range of the campaign. Specimens were tested for anti-measles virus and anti-rubella virus IgG antibodies with a different indirect EIA (Enzygnost; Siemens, Munich, Germany).^10^

Precampaign and postcampaign serosurvey results are presented as weighted seroprevalence estimates based on each survey design. Provincial seroprevalence estimates were calculated using poststratification by age and sex based on 2016 population estimates from the Zambian Central Statistics Office. Confidence intervals (CIs) are presented as 95% Wilson CIs. Categorical variables and age-specific seroprevalence estimates were compared using Rao-Scott χ^2^ tests.

A total of 1105 specimens from the precampaign serosurvey and 543 specimens from the postcampaign serosurvey were tested for MR IgG antibodies. The precampaign serosurvey included a higher proportion of males and participants 15 years and older than the postcampaign serosurvey (Table 1). During the precampaign serosurvey, 7% of participants were HIV-seropositive; however, the HIV serostatus was unknown for the postcampaign serosurvey.

**Table 1:** Study population characteristics found in precampaign and postcampaign serosurveys

	Precampaign (*N* = 1105), %	Postcampaign (*N* = 543), %	*P* value
Age groups			< 0.001
0–4 years	20.0	21.7	
5–9 years	17.4	26.9	
10–14 years	13.7	17.3	
15–19 years	12.0	7.4	
20–49 years	36.9	26.7	
Male (%)	49.8	44.5	0.06
Males in each age group			< 0.001
0–4 years	50.6	56.0	
5–9 years	50.5	48.5	
10–14 years	50.5	53.1	
15–19 years	50.2	46.3	
20–49 years	48.7	25.2	

Characteristics are presented as weighted percentages to assess whether differences remain after accounting for the serosurvey design, as designated by the *P* value.

The measles seroprevalence before the vaccination campaign was 77.8% (95% CI, 73.2–81.9), and it increased to 96.4% (95% CI, 91.7–98.5) among children younger than 15 years after the campaign (Figure 1). Among those 15 years and older, the measles seroprevalence increased, but not significantly, from 84.3% in the precampaign serosurvey (95% CI, 77.8–89.2) to 93.3% in the postcampaign serosurvey (95% CI, 84.5–97.3) (Figure 1).

**Figure 1.**
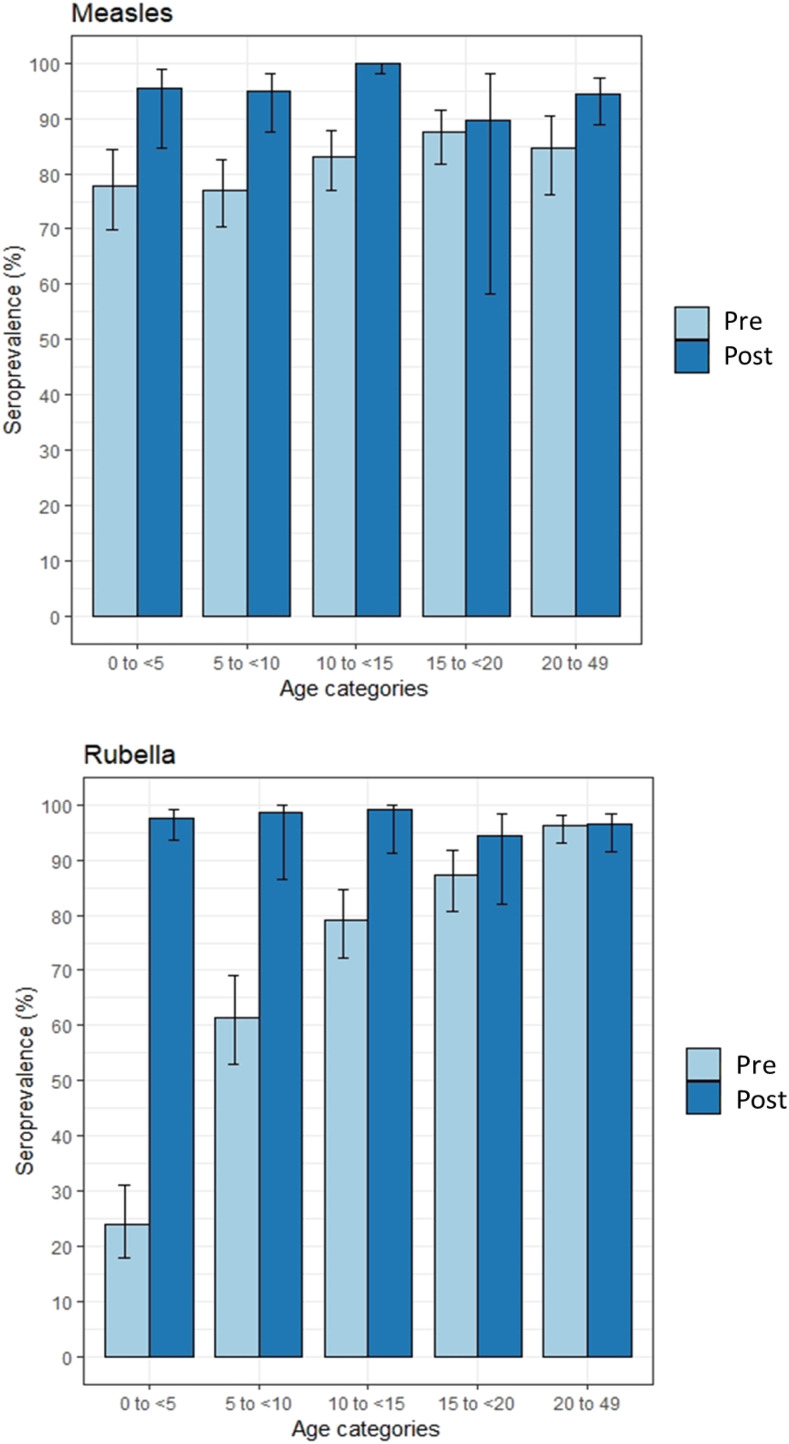
Seroprevalence by age for measles and rubella in the precampaign and postcampaign serosurveys. Light blue and dark blue lines represent the weighted seroprevalence estimates for the precampaign and postcampaign serosurveys, respectively. Taylor series 95% confidence intervals are at the top of each bar. Equivocal results were classified as seropositive. Weighting was based on the survey design. This figure appears in color at www.ajtmh.org.

The precampaign serosurvey showed that the measles seroprevalence was lower than expected based on vaccination coverage estimates. High first-dose measles coverage was reported in Southern Province by administrative data from the Ministry of Health (85% in 2013 to 2014, 100% in 2015, and 110% in 2016). Additionally, a second dose of measles vaccine was introduced in 2013, with vaccination coverage increasing from 42% in 2014 to 59% in 2016 (Zambian Ministry of Health, unpublished data). Zambia also conducted nationwide measles vaccination campaigns in 2002/2003, 2007, and 2012, with reported high coverage. Therefore, participants younger than 15 years had multiple opportunities for vaccination during both routine immunization and vaccination campaigns. Despite these vaccination efforts, precampaign seroprevalence was less than 80% among children younger than 15 years, thus demonstrating the need for the 2016 MR campaign targeting a wide age range.

The rubella seroprevalence increased from 51.3% (95% CI, 45.6–57.0) to 98.3% (95% CI, 95.5–99.4) among children younger than 15 years after the vaccination campaign (Figure 1). Before the campaign, the rubella seroprevalence had a strong positive association with age, increasing from 23.9% (95% CI, 17.8–31.2) among children younger than 5 years to 96.2% (95% CI, 93.0–98.0) among adults 20 to 49 years. This followed the typical rubella age-specific seroprevalence curve before vaccine introduction, whereby seropositivity results from natural infection and increases with age based on cumulative exposure to the virus.^11–13^

Conducting a campaign with a wide age range to introduce RCV substantially increased seroprevalence among children and closed the rubella immunity gaps. After the campaign, there was no statistically significant difference in rubella seroprevalence based on age, but there was slightly lower seroprevalence for the 15- to 19-year-old age group, who were just beyond the eligible age for the campaign. Immunity in this age group is important to prevent congenital rubella syndrome and is unlikely to be reached by future vaccination opportunities.^10,14^ Because the introduction of a rubella vaccine changes the age-specific population immunity dynamics, monitoring rubella vaccination coverage as well as case-based surveillance and seroprevalence, particularly among women of childbearing age, are important to track the risk of congenital rubella syndrome.

Serological surveys conducted before and after an MR vaccination campaign demonstrated that MR seroprevalence significantly increased after the MR vaccination campaign for the target age groups in Southern Province, Zambia. However, differences in the serosurvey methodologies make it difficult to attribute the changes solely to the MR campaign. These data were collected from two different cross-sectional serosurveys that both sub-sampled from larger studies conducted for different purposes with different survey designs. Both larger studies used the same 2010 census sampling frame. Although several differences between the two serosurveys were accounted for through weighting, the nonsignificant increase in measles seroprevalence among adults older than 15 years could not be explained by the vaccination campaign or measles outbreaks. Although not statistically significant, this difference may represent a selection bias or residual confounding because of differences in those who participated in the serosurveys. If there was differential participation of HIV-infected persons, then this could have affected measles seroprevalence estimates.^15^ However, we could not adjust the postcampaign serosurvey because information regarding the HIV infection status was unknown. Similarly, we could not determine whether this was attributable to differences in the diagnostic accuracy of the measles EIA kits. The Euroimmun EIA kit, used during the precampaign serosurvey, has been reported to have lower sensitivity than the Siemens Enzygnost EIA kit, and this difference may have contributed to underestimation of the seroprevalence during the precampaign period.^16^

The serosurvey conducted after the vaccination campaign identified high seroprevalence to MR, which was in line with the 96.8% vaccination coverage rate (95% CI, 94.7–98.1) reported for the 2016 campaign.^9^ Zambia has continued to report high first-dose coverage, but coverage with the second dose has stagnated at approximately 60%. Cases of MR have been reported since the MR campaign, but outbreaks have occurred primarily along the border with neighboring countries.^17^ To address the potential emerging immunity gap for children born after 2016, Zambia conducted a nationwide MR vaccination campaign in November 2020 to supplement routine immunization.

Although leveraging existing surveys can be challenging to assess the vaccination campaign impact, this study illustrated that the 2016 MR campaign substantially increased the MR seroprevalence. To our knowledge, this is the first analysis to use two serosurveys with blood specimens to estimate changes in seroprevalence after a vaccination campaign; however, this has been accomplished using oral fluid.^4,5,18^ Precampaign data are useful for understanding immunity gaps and establishing a baseline for estimating the impact of a campaign. Vaccination coverage estimates do not adequately highlight the significant impact of the mass vaccination campaign in 2016, and they could not have identified the slightly lower rubella immunity of 15- to 19-year-olds. Continued monitoring of population immunity can help identify the need for future targeted vaccination strategies.
